# Long-term stability of surgical-orthodontic correction of class III malocclusions with long-face syndrome

**DOI:** 10.4317/medoral.17647

**Published:** 2011-12-06

**Authors:** David Gallego-Romero, José M. Llamas-Carrera, Daniel Torres-Lagares, Vanessa Paredes, Eduardo Espinar, Eduardo Guevara, José L. Gutiérrez-Pérez

**Affiliations:** 1 Associate Professor – University of Seville; 2 Assistant Professor of Oral Surgery – University of Seville; 3 Associate Professor – University of Valence; 4 Master in Orthodontics – University of Seville; 5 Head Professor of Oral Surgery – University of Seville

## Abstract

Objectives: In the first place, to evaluate skeletal changes of the maxilla and mandible induced by surgical-orthodontic correction of malocclusions class III with long-face syndrome and secondly, to analyze the stability of these skeletal changes in the long term (more than 6 years). Design of Study: A retrospective, unicentric and longitudinal study of 19 patients who had undergone surgical and orthodontic therapy for class III skeletal malocclusion with long-face syndrome was undertaken. A cephalometric analysis based on 8 angle measurements, and statistical analyses at three different points in time (before orthodontic treatment, after orthognathic surgery and after a retention period of at least 6 years) were carried out. Results: The changes produced following surgery show that, with the exception of the maxillary plane and the facial axis, all other variables presented changes of great statistical difference. Conclusions: Skeletal changes after orthodontic-surgical correction present maxillary advance, mandibular regression and mandibular anterorotation. The angles that represent the mandibular vertical position (ramus angle, goniac angle and mandibular plane angle) showed statistically significant relapses and no stability in contrast to the facial axis.

** Key words:**Long term results, stability, relapse, orthognathic surgery, class III, long face.

## Introduction

An orthognathic surgery treatment represents a significant effort for both the patient and the professional and, in this sense, both aspire to see that effort rewarded with a result not only successful, but also reasonably stable over time (1).

Facial syndromes related to excessive vertical dimension, such as Class III malocclusion with long-face syndrome, can be treated by means of surgical manipulation of the jaws, so improving the skeletal-dental relationship and resulting in a more aesthetic proportion (1).

The occurrence of relapse has been widely studied to determine the factors affecting it; these include environmental factors such as masticatory function, a proper dental interdigitation, bone healing, condylar positioning in the glenoid cavity, neuromuscular adaptation, magnitude of surgical change, duration and method of maxillomandibular fixation, resorption and condylar growth, tongue pressure, patient age, surgeon experience or the use of bone grafts in maxillary breakthroughs. Sagittal problems are more stable after treatment, whereas vertical problems, which our study is concerned with, are less so (2-10).

Although many studies have shown stability of results following orthognathic surgery versus orthodontic treatment (11,12) few have reported such stability in mandibular anterorotation (13). Shendel and Epker (13) reported poor skeletal stability in mandibular anterorotation; they related this fact to the increase in posterior facial height, associated with an increase in the pterigo-maseteric musculature length. Proffit et al. (14) found that the surgical decrease of anterior facial height, caused by mandibular anterorotation in the anterior open bite correction, could compromise resulting stability. Their results showed, that, in patients with maxillary vertical excess and antero-posterior mandibular deficiency, the vertical repositioning of the maxilla and the mandibular advance were more stable with a clinical success rate of 60%. On the other hand, Chemello et al. (15) did report stable results in anterorotations, concluding that the success of these cases can be achievable through correct orthodontic treatment, proper surgical technique and the presence of healthy TMJs.

Despite the fact that the number of skeletal Class III patients with long-face syndrome who have undergone surgery has increased remarkably (16,17), it is surprisingly difficult to find data and reports on the long-term stability of this procedure with follow up results of more than 3 years after therapy has ended. This is, therefore, the reason for undertaking this study. Furthermore, the long term results of surgical procedures are not well documented for sufficiently homogeneous samples of patients with increased lower vertical facial height and skeletal Class III.

The objectives of this study were, in the first place, to evaluate skeletal changes of the maxilla and mandible induced by surgical-orthodontic correction of malocclusions class III with long-face syndrome, and secondly, to analyze stability of these skeletal changes after a long retention period (more than 6 years).

## Material and Methods

This study is a retrospective, unicentric, longitudinal clinical study of skeletal Class III patients with long-face syndrome. The study population was defined as long face syndrome based on cephalometric values. All patients were cephalometrically and facially dolichofacial (proportions of facial thirds and mandibular plane severely rotated clockwise). All the patients had anterior open bite before treatment.

The aims of the treatment were both, to shorten face height and to close open bite. All patients had previously undergone pre-surgical orthodontic treatment and surgical correction afterwards through bimaxillary surgery. All cases were operated on by two surgeons who operate jointly on all their patients. Intermaxillary fixation was used in the surgeries.

Included in our sample were both male and female patients who had obtained good results in facial and occlusal parameters after the treatment and whose clinical records were valid and available for conducting a long-term follow up of at least 6 years following the end of the surgical-orthodontic treatment. A further inclusion criterion was the presence of stability in vertical and sagittal dentocclusal landmarks after the same period of time, no more than 2 mm of relapse in overbite or overjet being considered as stable. We eliminated the cases with little dental stability, as the aim was to study whether skeletal stability had accompanied dental stability. Those patients who did not fulfill the inclusion criteria and those whose radiographic records presented poor quality or heterogeneous magnifications were excluded from the study.

19 patients participated in this study; 14 female (73.7%) and 5 male (26.3%). The cephalometric analysis applied in the present study was a variation of the method developed by Bjork and Skieller which was modified by Nielsen. A total of 8 angle measurements were used at three different points in time (before orthodontic treatment, after orthognathic surgery and after a retention period of at least 6 years). All images were recorded by the same operator and machine (Ortoceph 50®, Siemens). All selected radiographs fulfill the following quality requirements: recorded in the maximum intercuspal; correct visualization of required bone structures; no magnification problems and, in order to obtain a valid superposition, the cephalostate was used correctly to avoid duplicated images resulting from head rotation.

**Figure 1:**
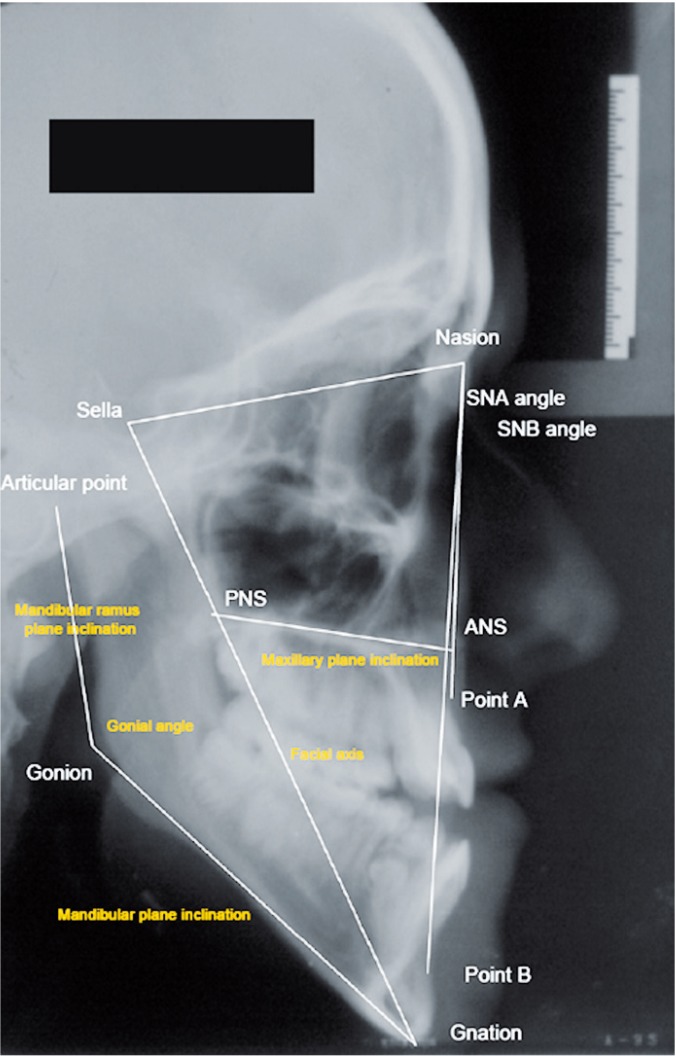
Angle values used in our study.

The cephalometric landmarks shown in  Table 1 were identified in the lateral cephalograms and angle values were determined (Fig. 1).

**Table 1:**
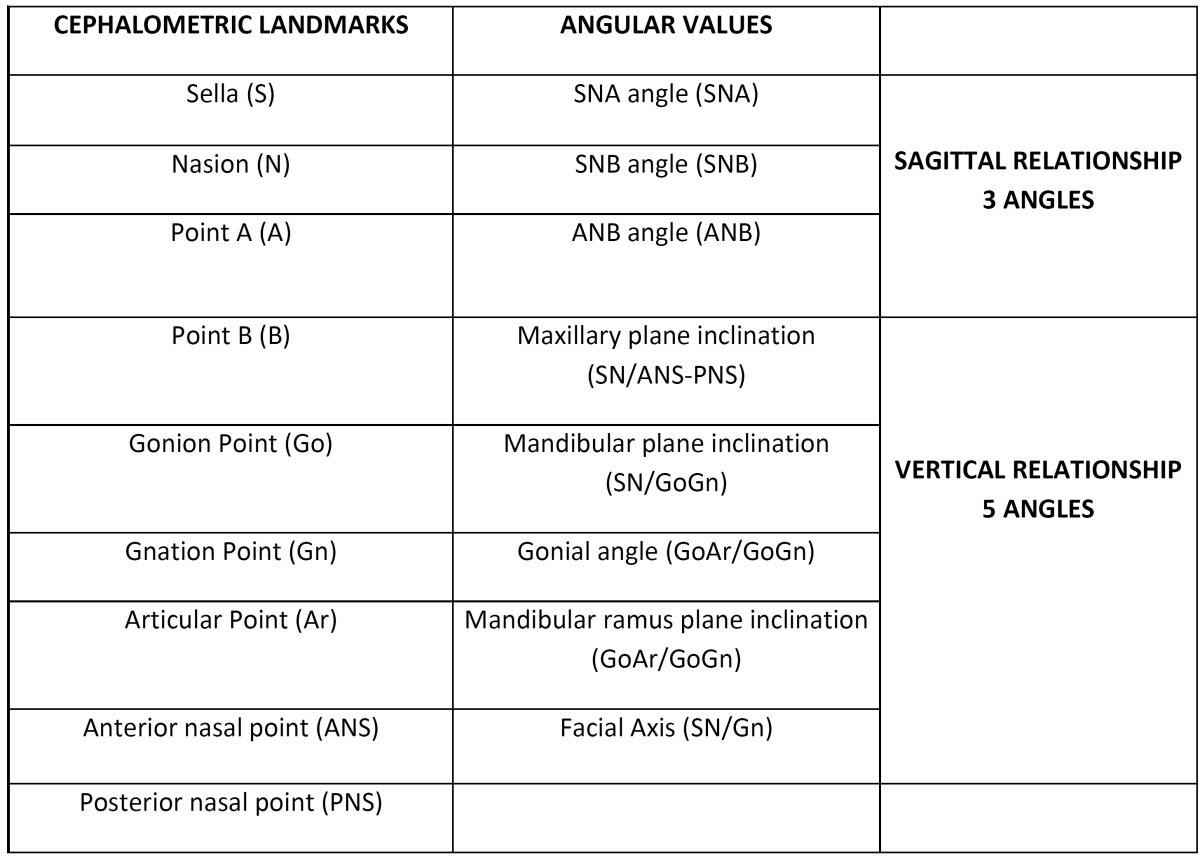
Cephalometric landmarks and angular values.

Anterior skull base length was considered as the plane of reference, represented by the Sella-Nasion line (SN). The ANB angle was preferred instead of ANPg, as some of the patients had undergone genioplasty procedures.

The data studied were appropriate as they allowed us to assess the positional changes in the maxilla, both sagittally (SNA) and vertically (maxillary plane), in the mandible, both sagittally (SNB) and vertically (mandibular plane, mandibular angle, ramus angle and facial axis) and the intermaxillary sagittal relationship (angle ANB).

**Figure 2:**
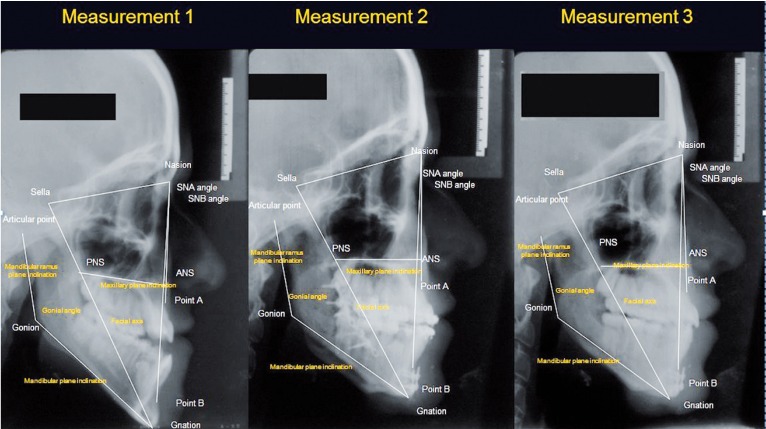
Cephalometric tracings at the three observations.

These measurements were carried out at three different stages of the study: The 1st set of measurements was recorded prior to orthodontic treatment, the 2nd after the end of surgical-orthodontic treatment and the 3rd after a retention period of at least 6 years. Figure 2 shows an example of these measurements taken on one patient at the three different stages of the study. The mean time period elapsing between the 1st and 2nd measurement was 2.2 years (range: 1-3.5 years), between the 2nd and 3rd 7.6 years (range: 6-10 years) and finally, between the 1st and the 3rd measurement 9.8 years (range: 8-12 years).

All patients were treated with the same surgical and fixation technique. Good interincisal contact was obtained. Two surgeons working together operated on all cases. In order to validate the measurement procedure, we carried out error assessment on ten randomly selected tracings, which were measured once again. Student’s t-test and correlation did not show significant differences (p > 0.05 and r > 0.95). We also carried out nonparametric Wilcoxon Signed Rank test to determine the significance of the changes observed when comparing the three stages of measurements. A p value of < 0.05 was considered statistically significant. All statistics were analyzed using Statview 4.5 for Macintosh.

## Results

The age differences between males and females were statistically significant at the 1st and 2nd measurements ( Table 2).

**Table 2:**
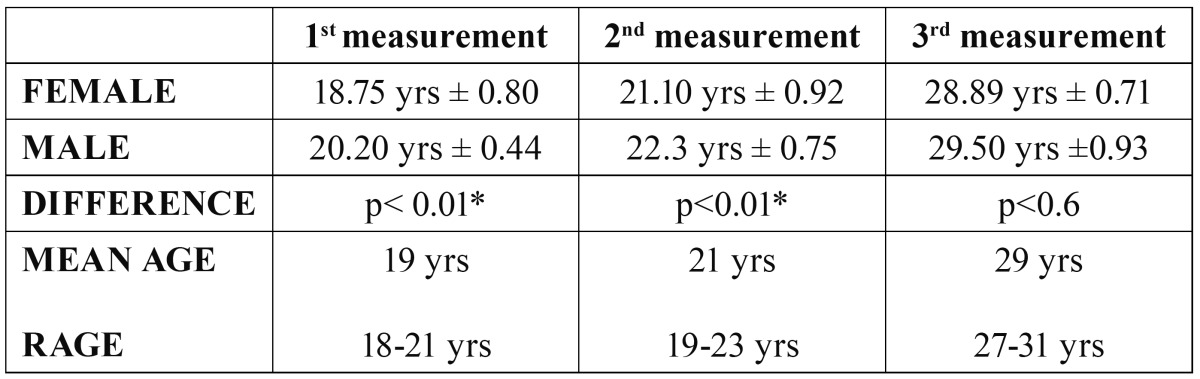
Ages for males and females in the 1st, 2nd and 3rd measurements.

The data obtained are shown in  Table 3. From the 1st to the 2nd, we observed variations resulting from the treatment, while changes observed from the 2nd to the 3rd reflect the long-term results of the variables under study. Finally, the changes observed between the 1st and the 3rd represent the magnitude of total changes, whether relapse has taken place or not.

**Table 3:**
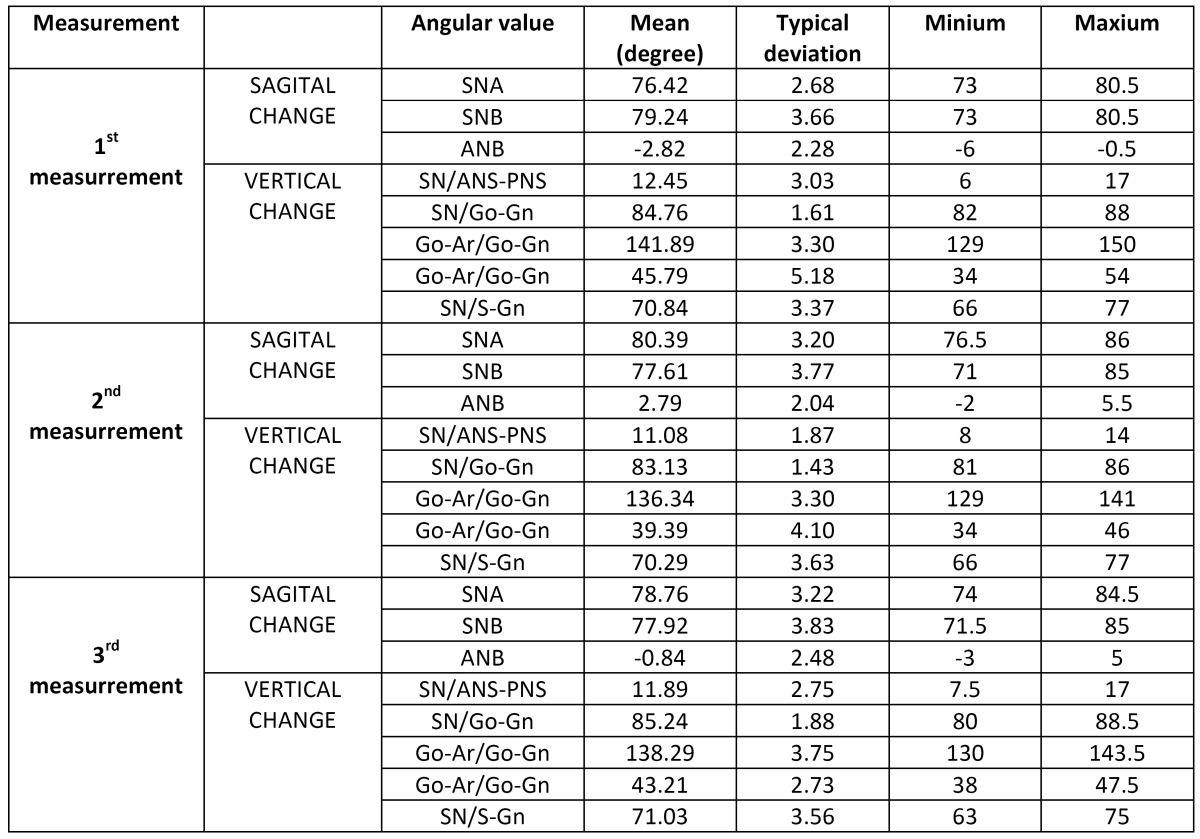
Angular values of the sample at the different measurement points(1st, 2nd and 3rd measurement).

The statistical significance of the differences and/or changes shown by the data is presented in table 4. Statistically significant p values less than 0.001 are marked as ***, between 0.001 and < 0.01 as **, and between 0.01 and < 0.05 as *. A “p” value higher than 0.05 is not statistically significant (NS).

Our patients had a retruded sagittal position of the upper jaw with respect to the cranial base (SNA 76.42º ± 2.68º), while the angle SNB was within the normal range (79.24º ± 3.66º). The maxillary plane (12.45° ± 3.03º) showed a slight anterior maxillary decrease while the mandibular plane increased. The mandibular angle (141.89° ± 3.30º) indicates the verticality of the mandibular ramus while the reduced ramus plane (84.76° ± 1.61º) is characteristic of an excess of vertical mandibular growth and occurs mainly due to an opening of the mandibular body at the mandibular angle. Finally, the facial axis (70.29° ± 3.63º) showed a greater opening than normal.

In the 2nd measurements, the angle SNA approached values closer to normal (p<0.0001) although the SNB was 77.61° ± 3.77º (p <0.0001). Although in the cases of the maxillary plane (11.08 ° ± 1.87º, P <0.01), of the mandibular plane (-13.95%, p<0.0001) and the ramus plane (83.13° ± 1.43º, p <0.0001), normal and statistically significant values are approached, they do not quite reach normal ones.

The changes occurred in the ramus plane (increased 2.53 º, + 2.11%, which, despite its lack of statistical significance, presents a loss of 130% in the reduction achieved after surgery), the mandibular angle (+35.13% of the reduction obtained after surgery, p<0.0001) and the mandibular plane (increase of 3.82º, a loss of almost 60% reduction after surgery, p <0.0001).

## Discussion

On analyzing the literature we noticed how difficult it is to combine the three most important aspects, i.e. homogeneity, sample size and lengthy follow-up period in these types of studies. Studies with lengthy follow-up periods and a homogeneous sample tend to be of small sample size, whereas larger samples sizes are usually more heterogeneous with a shorter follow-up period.

19 patients participated in this study; 14 female and 5 male. Both the clinical and statistical results obtained have demonstrated the value and quality of this sample for obtaining statistically significant results.

Thus, in contrast to the most significant characteristic of the present study, the mean follow-up period (7.6 years), and the specific nature of the sample (class III long-face syndrome), most studies dealing with these aspects include samples of no more than 20 patients (17,18). Other studies with samples of more than 20 patients are not so sample-specific (9) or have a mean follow-up period of less than 6 years (19,20).

Our patients had a normal SNB angle due to mandibular posterorrotation that usually accompanies class III malocclusion and causes point B to occupy a lower and posterior spatial location. The decrease of the maxillary plane and the increase of the mandibular plane can be interpreted as a mandibular posterorrotation, this being logical in dolichofacial patients. The facial axis indicates once again the characteristic posterorrotation of dolichofacial patients. In the 2nd measurements, the angle SNB was still outside the range of normal reference. In our view, this value has been affected more by the sagittal retrusion of point B following mandibular regression than by the advance of the same due to anterorotation. Hoppenreijs et al. (21) found, however, an increase of 2.74º in the SNB. After surgery, the angle ANB increased 5.61° (+198.93%, p <0.0001), results similar to those of Costa et al. (22), who found an increase of 5.47º.

The mandibular angle decreased by 3.91% (p <0.0001), a surgical anterorotation of the distal segment after osteotomy taking place. Moreover, the facial axis decreased by -0.55º, being the smallest change (-0.77%) without statistical significance or clinical repercussions. In short, the overall analysis of changes after surgery shows that, except for the maxillary plane and facial axis, all other variables present highly statistically significant changes. Thus, changes in the parameters show a sagittal advance of the maxilla (increased SNA), a sagittal mandibular regression (reduced SNB) and mandibular anterorotation (decreased level of the branch, the mandibular angle and mandibular plane). Similarly, the angle ANB increases and adequately situates the maxilla in relations to the mandible.

Regarding the changes of values between the 2nd and 3rd observation, i.e. the loss of the progress made with surgery in the long term, our data indicates that, in general, there was a relapse of great clinical and statistical significance in some of the cephalometric parameters studied relating to the vertical characteristics of the sample: on the ramus plane, on the mandibular angle and on the mandibular plane. These data express a return of the jaw to parameters that reflect their posterorotation and verticality. However, there is not such a marked relapse in terms of the sagittal position of the mandible (SNB, with a change of barely 0.41% in its value and 19.63% in the surgery¬induced change) and its relationship to the maxilla. Authors such as Costa et al. (22) found an increase of 0.92° in a year (42.59% of the change achieved with surgery, -2.16°), while Dowling et al. (23) found no change in the SNB after 3 years, although they were only considering the upper jaw. While the change in ANB is statistically significant (reduced by 1.95°, -69.89% of the absolute value of the parameter, p <0.01), this is caused more by the loss of the sagittal location of point A than by point B (SNA presents a significant variation in this comparison, something which does not occur in SNB). The value of this change after monitoring shows again a position of abnormality with regard to the intermaxillary sagittal relationship, with the maxilla located roughly on the same sagittal level as the mandible. In this, our study is in agreement with those of Dowling et al. (23) and Costa et al. (22). Finally, changes in the maxillary plane or facial axis are clinically negligible, although the former presents statistical significance while the latter does not. It may, therefore, be stated that there is little variation in facial axis, which is still above normal values, reflecting the dolichofacial pattern. If we analyze the changes that have taken place with regard to the initial situation in our sample, despite the losses in the postoperative period, we find that, with the exception of the maxillary plane and facial axis, all other variables showed statistically significant changes, some to a greater extent than others. The sagittal location of the maxilla retains a significantly favorable change (p <0.0001), thanks to improved sagittal position of the maxilla (increase of the SNA, +3.06%, p <0.01) and of the mandible (SNB reduction, -1.26%, p <0.01). As a result, the intermaxillary sagittal relationship (ANB) maintains a change of maximum statistical significance (+129.78%, p <0.0001) and, although not within the parameters of absolute normality, it does manage to situate the maxilla on the same sagittal level as the mandible. Similarly, the parameters relating to anterorotation correction of the mandible still show a change of great significance (the ramus plane (-5.63%, p <0.01), the mandibular plane (+0.55%, p <0.0001) and the mandibular angle (-2.54%, p <0.0001). These changes are in line, but to a lesser extent, to that achieved after surgery in the correction of skeletal Class III malocclusion and dolichofacial pattern. Therefore, the changes in the parameters demonstrate an advance of the maxilla, a mandibular regression and an anterorotation of the mandible. Even though the changes are statistically significant, they are not clinically significant. Finally, we have to keep in mind that the sample is small and that results cannot be generalized.

In conclusions, 1.- Skeletal changes after orthodontic-surgical correction present maxillary advance, mandibular regression and mandibular anterorotation, and, 2.- The three angles that represent the mandibular vertical position (ramus angle, goniac angle and mandibular plane angle) showed statistically significant relapses and no stability in contrast to the facial axis.
